# Charcot arthropathy: The diagnostic dilemma of a painless, destructive joint

**DOI:** 10.1002/ccr3.2603

**Published:** 2019-12-11

**Authors:** Nikiforos Galanis, Aikaterini Kyriakou, Ioannis Delniotis, Paraskevi Charanidi, Paraskevas Mavropoulos, James Inklebarger

**Affiliations:** ^1^ Aristotle University School of Medicine Thessaloniki Greece; ^2^ Orthopadische Klinik Volmarstein, Kinderorthopädie & Neuroorthopädie Wetter Germany; ^3^ NHS London HealthShare Ltd Kent UK

**Keywords:** charcot, diabetes mellitus, painless joint

## Abstract

The impressive clinical picture of Charcot joint reflects a high energy trauma injury which in the contrary is a progressive, painless arthropathy. Early and accurate diagnosis is crucial. A favorable outcome is elicited when joint is treated promptly, while late or misdiagnosis may lead to amputation.

## Graphical abstract

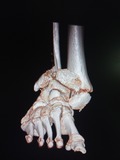

A 49‐year‐old male presented at the emergency room with a painless and deformed right ankle. The patient reported that the loss of function was due to a low energy injury. He described that he had suddenly experienced a feeling of unstable joint when he tried to get off the pavement. He declared no significant medical history and received no medications regularly. Upon clinical examination, joint deformity, ligaments deficiency, bony prominence, and edema were found. Moreover, erythema located on the ankle was present. The initial laboratory workup revealed increased levels of glucose (318 mg/dL) and no other significant findings. The patient was tested negative for a number of infective diseases (HIV, hepatitis, syphilis, QuantiFERON test, fungal, and bacterial culture). Potential causes of peripheral neuropathy were investigated, and levels of HbA1c were increased. Imaging revealed distraction and resorption of the ankle joint (Figures 1, 2) and excluded syringomyelia. Based on the typical clinical presentation, the diagnosis of Charcot joint was made.^1^, ^2^ The patient underwent arthrodesis with Ilizarov external fixation and with a satisfactory, one‐year outcome (Figure 3). Oral diabetes medication was also initiated, and glucose level was controlled. The impressive clinical picture of Charcot joint reflects a high energy trauma injury which in the contrary is a progressive, painless arthropathy.^1^, ^2^ Even though it is reported to be not so rare,^1^, ^2^ a wide range of clinicians seems to ignore this entity. Early and accurate diagnosis is crucial. A favorable outcome is elicited when joint is treated promptly, while late or misdiagnosis may lead to amputation.^1^, ^2^

**Figure 1:**
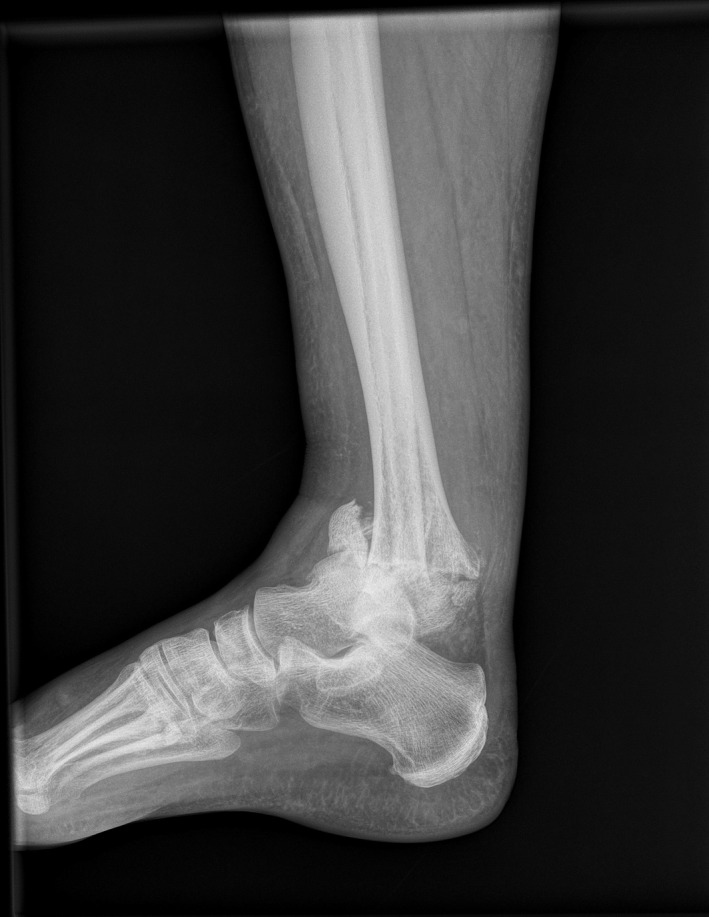
The paradox of a dislocated, painless ankle joint

**Figure 2:**
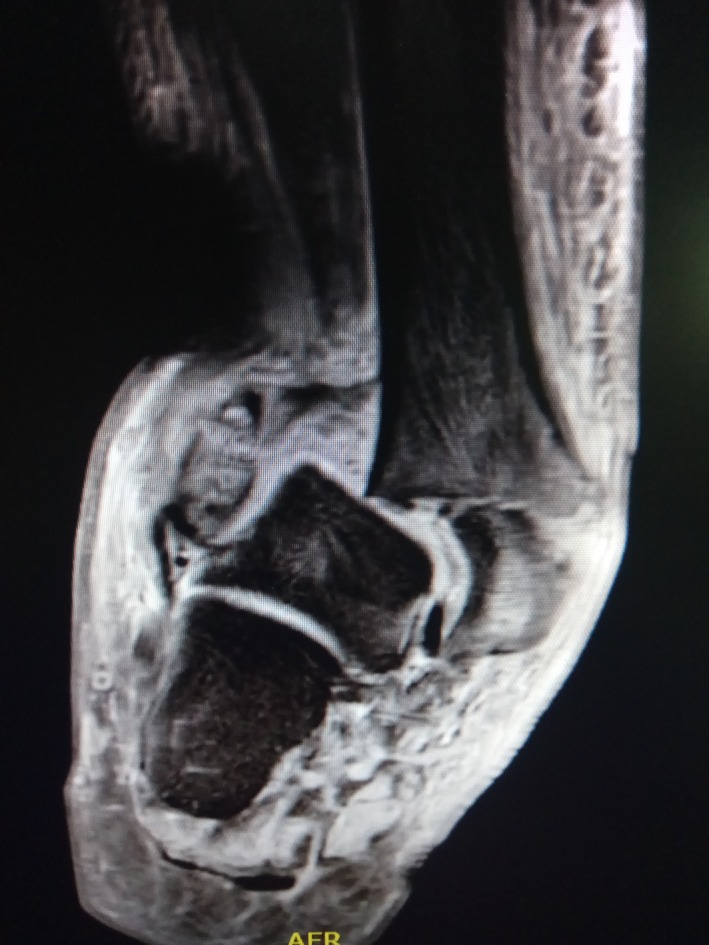
MRI of Charcot joint

**Figure 3:**
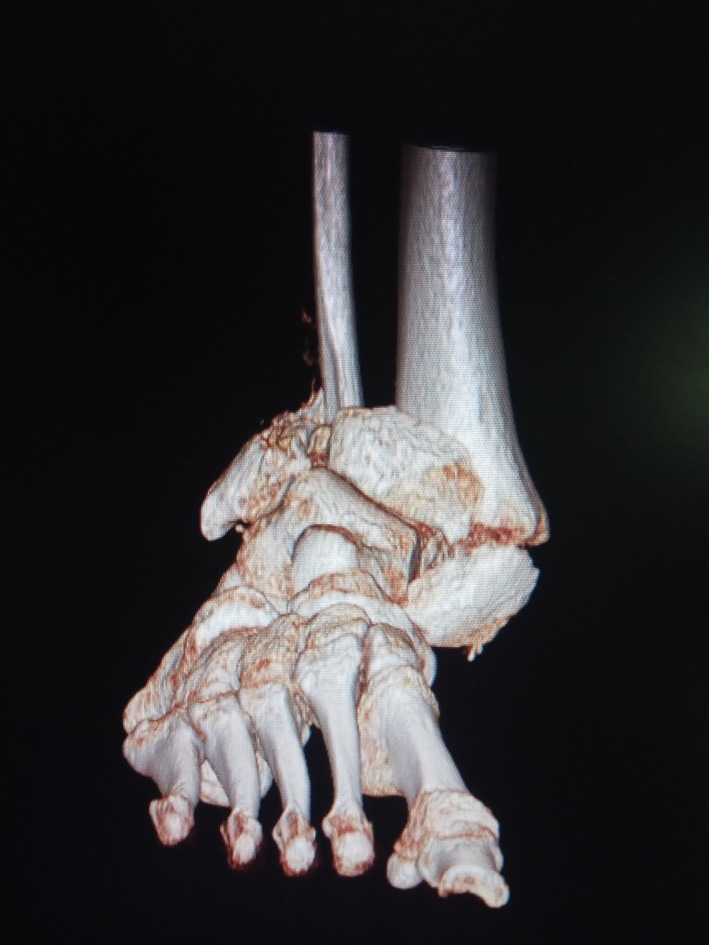
CT reconstruction of Charcot joint

## CONFLICT OF INTEREST

None.

## AUTHOR CONTRIBUTIONS

NG: drafted the manuscript, obtained the photographs, and contributed to patient care. AK: drafted the manuscript. ID and JI: critically reviewed the paper. PC and PM: contributed to patient care and reviewed the manuscript.
